# Arterial stiffness and hypertension

**DOI:** 10.1186/s40885-018-0102-8

**Published:** 2018-12-01

**Authors:** Young S. Oh

**Affiliations:** Vascular Biology and Hypertension Branch, Division of Cardiovascular Sciences, National Heart, Lung, and Blood Institute (NHLBI), National Institutes of Health (NIH), 6701 Rockledge Drive, Room 8106, Bethesda, MD 20892 USA

**Keywords:** Hypertension, Arterial, Aortic stiffness, Cardiovascular disease, Vascular biology

## Abstract

Measures of the functional and structural properties of blood vessels can be used to assess preclinical stage of vascular disorders. Recent experimental and population studies show that arterial stiffening precedes development of high blood pressure, and can be used to predict future cardiovascular events. Arterial stiffness was also shown to be reversible in several experimental models of various conditions. Since reversing arterial stiffness could prevent development of hypertension and other clinical conditions, understanding the biological mechanisms of arterial stiffening and investigating potential therapeutic interventions to modulate arterial stiffness are important research topics. For research and application in general clinical settings, it is an important step to develop reliable devices and a standardized arterial stiffness measurement protocol.

## Introduction

The walls of large arteries, especially the aorta, lose elasticity over time, and this process results in increased arterial stiffness. Arterial stiffening, at least in part, reflects gradual fragmentation and loss of elastin fibers and accumulation of stiffer collagen fibers in the arterial wall [1]. Increased arterial stiffness is closely linked to increased risk of hypertension and other diseases, such as chronic kidney disease and stroke [2]. In this brief review, I will discuss recent progress in relating arterial stiffness research to hypertension.

## Arterial stiffness precedes hypertension

Although the causality between increased arterial stiffness and hypertension is complex because of many confounding factors (e.g., aging, diet, concurrent disease, life style, etc.), recent studies in humans and animals suggest that increased arterial stiffness can precede hypertension. For example, several research projects funded by the NHLBI (National Heart, Lung, and Blood Institute) – the NIH (National Institutes of Health) Institute focused on supporting cardiovascular research – had examined the temporal and causal relationship between arterial stiffness and hypertension [3]. Studies in five different animal models concluded that arterial stiffness precedes high blood pressure. These animal models included: (i) diet-included obesity model, (ii) elastin gene knock-out model, (iii) stroke-prone Dahl salt-sensitive rat model, (iv) klotho gene knock-out model, and (v) type 2 diabetes model. In clinical studies, a consistent temporal sequence of arterial stiffness preceding hypertension was also observed in the Framingham Heart Cohort Study [4]. However, the biological mechanisms and cellular processes whereby increased arterial stiffness alone can lead to hypertension are still not understood, encouraging further investigation.

## Is arterial stiffness reversible?

Both human and animal studies have suggested that arterial stiffness is reversible. In a murine model of diet-induced obesity, the increased pulse wave velocity (PWV: the gold standard in vivo measure for arterial stiffness) in obese mice fed a high fat/high sucrose diet (HFHS) for 5 months was reduced to normal after returning obese mice to standard chow for 2 months [5]. During the 2-month period, indices of metabolic impairment of obese mice such as body weight, fat mass and hyperinsulinemia, returned to normal; PWV and high blood pressure also returned to normal. Further, Fry et al. [6] studied the potential effect of dietary resveratrol on arterial stiffness. The authors found that resveratrol, a polyphenol known to activate the deacetylase sirtuin-1, prevented the HFHS-induced inflammation and excess oxidant production in the arterial wall as well as the accompanying increase in PWV. Interestingly, administration of a sirtuin-1 specific activator (SRT1720), after 8 months of HFHS, decreased PWV to normal values within 2 weeks. The positive effect of dietary resveratrol on arterial stiffness was further replicated in non-human primates that were fed high caloric diets [7], underscoring its translational potential in humans.

Using an aging rat model (i.e., 20 month-old), Steppan et al. [8] studied the relationship between exercise, tissue transglutaminase (TG2) activity, and arterial stiffness; TG2, an enzyme catalyzing protein cross-links, is known to play a role in vascular stiffness with age [9]. The authors found that there was significant suppression of an age-associated increase in TG2 activity when animals were subjected to moderate-intensity exercise, which was correlated with increased nitric oxide bioavailability and reduced collagen depositions in the extracellular matrix. Interestingly, these biochemical changes did not translate into a significant alteration in vascular stiffness, supporting the hypothesis that once formed, the TG2 crosslinks may have a long half-life in the vascular matrix. Thus, it seems that the reversibility of vascular stiffness may be limited to a certain stage or type of vascular condition leading to stiffness.

In humans, short-term aerobic exercise (3 months) reduced arterial stiffness in older adults (> 65 years) with type 2 diabetes and might thereby lower the risk of cardiovascular morbidity and mortality [10]. A recent randomized clinical trial study (SAVE: Slow Adverse Vascular Effects of excess weight) also showed the reversibility of vascular stiffness by moderate-to-vigorous physical activity in overweight or obese young adults [11]. In addition, some anti-hypertensive medications (i.e., angiotensin converting enzyme inhibitor or angiotensin II receptor I antagonist) are shown to reduce arterial stiffness significantly [12]. Thus, arterial stiffness associated with some medical conditions can be reversed by life style change or treatment.

## Conclusion and perspectives

Arterial stiffness is an important arterial phenotype and an excellent indicator of cardiovascular morbidity and mortality [13]. It is an independent predictor of hypertension and cardiovascular diseases. Recent studies in animal models showed that large artery stiffening preceded development of high blood pressure. This temporal sequence was also observed in clinical studies. Nevertheless, it should be kept in mind that the relationship between arterial stiffness and blood pressure can be complex. For example, there are patients who have high blood pressure with normal PWV values [14].

**Fig. 1:**
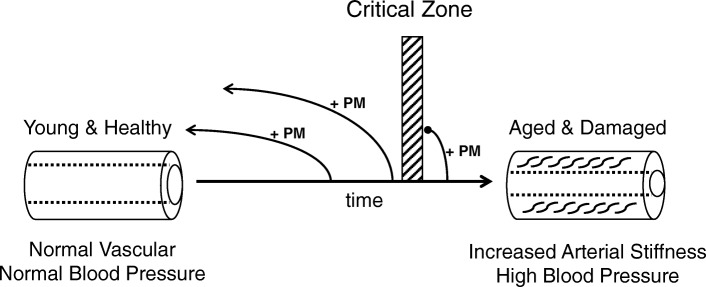
A simplified model of arterial stiffening and its reversibility. With aging, blood vessel structural changes and endothelial dysfunction can occur. Various factors contribute to arterial stiffening, such as changes in the composition of elastin and collagen fibers, calcification, and inflammation in the arterial wall. It seems there is a critical time zone during the process of arterial stiffening when a PM (Positive Modifier: such as exercise, healthy diet, weight loss, or anti-hypertensive drug) cannot reverse vascular stiffness. Future research is needed to characterize this critical zone

Both arterial stiffness and hypertension are positively associated with aging. Studies from animals and humans suggest that arterial stiffness can be reversible under certain conditions (Fig. 1). Niiranen et al. [15] have recently studied healthy vascular aging (HVA) - defined as absence of hypertension and lack of arterial stiffness – in more than 3100 participants (aged *>* 50 years) of the Framingham Heart Study and have found that maintaining HVA beyond age 70 is extremely challenging. With rapid population aging, it will be important in the future to explore the possibility of prevention or reversal of arterial stiffness as a potential therapeutic strategy to control hypertension and/or hypertension-related diseases. In this regard, the European Society of Hypertension and the European Society of Cardiology published a guideline in 2013 to suggest the measurement of arterial stiffness as a way of evaluating hypertensive patients at high cardiovascular risk [2]. In recognizing the clinical importance of arterial stiffness, the American Heart Association also published a scientific statement to encourage further improvement and standardization of arterial stiffness measurements for clinical use and vascular research [13]. Once a standardized measurement protocol and reliable devices are available, arterial stiffness can provide us valuable information about the risk of hypertension, cardiovascular disease, and early vascular aging.
